# Pseudogene *RACGAP1P* activates RACGAP1/Rho/ERK signalling axis as a competing endogenous RNA to promote hepatocellular carcinoma early recurrence

**DOI:** 10.1038/s41419-019-1666-2

**Published:** 2019-06-03

**Authors:** Meng-Yao Wang, Dong-Ping Chen, Bin Qi, Ming-Yi Li, Yan-Yi Zhu, Wen-Jing Yin, Lu He, Yi Yu, Zhou-Yu Li, Ling Lin, Fang Yang, Zhi-Rui Lin, Jin-Quan Liu

**Affiliations:** 1 Department of Radiation Oncology, Affiliated Cancer Hospital and Institute of Guangzhou Medical University, 510245 Guangzhou, China; 2 State Key Laboratory of Oncology in South China, Collaborative Innovation Center for Cancer Medicine, Sun Yat-sen University Cancer Center, 510245 Guangzhou, China

**Keywords:** Tumour biomarkers, Cancer

## Abstract

Accumulating evidence has indicated crucial roles for pseudogenes in human cancers. However, the roles played by pseudogenes in the pathogenesis of HCC, particularly HCC early recurrence, still incompletely elucidated. Herein, we identify a novel early recurrence related pseudogene *RACGAP1P* which was significantly upregulated in HCC and was associated with larger tumour size, advanced clinical stage, abnormal AFP level and shorter survival time. In vitro and in vivo experiments have shown that *RACGAP1P* is a prerequisite for the development of malignant characteristics of HCC cells, including cell growth and migration. Mechanistic investigations indicated that *RACGAP1P* elicits its oncogenic activity as a ceRNA to sequestrate *miR-15-5p* from its endogenous target *RACGAP1*, thereby leading to the upregulation of RACGAP1 and the activation of RhoA/ERK signalling. These results may provide new insights into the functional crosstalk of the pseudogene/miRNA/parent-gene genetic network during HCC early relapse and may contribute to improving the clinical intervention for this subset of HCC patients.

## Introduction

Hepatocellular carcinoma (HCC) is one of most frequent cause of cancer-related mortality worldwide, with more than half a million deaths per annum^1,2^. Early HCC recurrence is predominantly responsible for dismal outcomes and is the major obstacle to improving HCC patients’ survival^3,4^. Therefore, molecular investigation of the early recurrence of HCC is the major challenge and there is a pressing need to develop new biomarkers to screen out HCC patients with the higher risk of recurrence, who would be extremely beneficial for the clinical intervention^5^.

In pursuit of the prediction of early recurrence for HCC patients, previous studies have highlighted valuable biomarkers such as *Rac GTPase Activating Protein 1 (RACGAP1)*^6^, *Epithelial Cell Transforming 2* (*ECT2)*^7^, *Protein Regulator Of Cytokinesis 1 (PRC1)*^8^ et al., which are all protein-coding genes. Considering long non-coding RNAs (lncRNAs) make up the major component of human transcriptome, it is essential to determine whether lncRNAs may serve as diagnostic markers for HCC early relapse. Pseudogenes, defined as dysfunctional copies of protein-coding genes, represent an intriguing class of lncRNAs and were primitively considered as “genomic junk”, but their well preservation during the evolutionary pressures imposed by natural selection argues that they may possess important functions and that their dyregulation could contribute to the development of diseases^9,10^. Indeed, mounting evidence has suggested that pseudogenes are playing key roles in oncogenesis or tumour suppression^11,12^. In HCC, several lines of evidence have indicated that pseudogenes are aberrantly expressed, providing new insights into the pathogenesis of HCC^13–15^. However, few HCC early recurrence associated pseudogenes have been characterised thus far.

In the current study, we first identified a novel HCC early recurrence associated pseudogene *RACGAP1P*, of which the biological function and expression pattern in cancer have not been illuminated. We found that *RACGAP1P* was significantly upregulated in HCC tissues and markedly upregulated in the HCC samples with early recurrence. In addition, high expression of *RACGAP1P* in HCC was associated with poor prognosis. Mechanistically, *RACGAP1P* acts as an endogenous sponge for *miR-15-5p* to prevent its association with *RACGAP1*, leading to activation of the RhoA/ERK signalling axis and enhancing liver cancer cell proliferation and migration. Collectively, the results of this study show that *RACGAP1P* is an oncogenic regulator of HCC early recurrence, and may promote the development of effective recurrence-targeted therapy and improve the overall prognosis of HCC patients.

## Materials and methods

### Clinical specimens

Tumour and normal tissues from HCC patients that were histopathologically and clinically diagnosed, and did not receive neo-adjuvant treatment were obtained from the Affiliated Cancer Hospital and Institute of Guangzhou Medical University in Guangzhou. Written informed consent was obtained from each patient, and the study was approved by the Institute Research Ethics Committee at the Cancer Center.

### Cell culture, proliferation assays and colony-formation assays

The human cancer cell lines were maintained in Dulbecco’s modified Eagle’s medium supplemented with 10% FBS at 37 °C and 5% CO_2_.

For the proliferation assays, 1000 cells/200 μl of medium were plated in a 96-well plate (Corning) in six copies and stained with MTS (Sigma, M2128) for 3.5 h determined at OD_570_ with a microplate reader every day for 6 days.

For the colony-formation assays, 500 cells/2 ml were seeded into a 6-well plate (Corning) in triplicates. The cells were washed with phosphate-buffered saline (PBS), fixed with methanol for 10 min at room temperature, and stained with 1% crystal violet for 20 min. The colonies were counted after 10 days.

### Migration assay

For migration assays, 3 × 10^4^ HCC cells in 200 μl of serum-free DMEM were seeded into 24-well plates upper chambers with an 8-mm pore size filter (Falcon), the lower chamber was filled with 600 μl of 10% FBS–DMEM. After 24 h of incubation, the cells on the lower surface of the filter were fixed and stained with 1% crystal violet. The number of migrated cells in five random optical fields (×100 magnification) from triplicate filters was averaged.

### Animal experiments

All protocol was approved by the Institutional Animal Care and Use Committee of the Guangzhou medical University. For the tumourigenicity studies, Hep3B cells stably transfected with vector or *RACGAP1P-3’UTR* (1 × 10^6^ cells/tumour in 150 mL DMEM with 25% matrigel) were subcutaneously injected into either side of flanks of the 5 female BALB/c athymic nude mice (3–4 weeks of age). Tumour diameters were measured in indicated day after tumours were established. Finally, the mice were euthanized, and the tumours were isolated and weighed, the tumour volume was calculated as volume = length × width^2^ × 0.52.

### RNA extraction and real-time PCR

For real-time PCR, total RNA was extracted from cultured cell lines using Trizol reagent (Invitrogen) and subjected to reverse transcription using a cDNA Synthesis Kit (Thermo, K1622). Real-time PCR was performed using a SYBR FAST Universal qPCR Kit (KAPA, KK4602). The relative expression of the target mRNAs were calculated as two power values of ΔCt (the Ct of β-actin minus the Ct of the target cDNA). The sequences of the PCR primers used for amplification were as follows:

*β-actin* forward, 5′-AAGGTCATCC CTGAGCT GAA-3′;

*β-actin* reverse, 5′-TGACAAAGTG GTCGTTG AGG-3′;

*RACGAP1* forward, 5′-GAAAGCAGAGACTGAGCGAAG-3′;

*RACGAP1* reverse, 5′-GTTGAATGCTGCCAGATGTGT-3′.

*RACGAP1P* forward, 5′-GCACCTGTACTCTCTGCTCTAC-3′;

*RACGAP1P* reverse, 5′-TCCCAACAGTGACCAGAACA-3′.

*DICER1* forward, 5′-TGGATAGTGGGATGTCACTGG-3′;

*DICER1* reverse, 5′-CTCTGACCTTCCCGTCGTAAG-3′.

### Small interfering RNA transfection

The negative control small interfering RNA (NC) and siRNA targeting human DICER1 are 5′-CAGCACCTTTACCCTTAGT-3′, were purchased from GenePharma, transient transfections of HCC cells were performed by using the Lipofectamine RNA iMAX Reagent (Invitrogen) protocol with 30 pmol siRNA in Opti-MEM Medium (Invitrogen) were mixed, and incubated at room temperature for 5 min, then the mixture was added to the cells.

### MS2-RIP

The MS2bp-MS2bs-based RIP assay was performed according to previous reports^16^, with modifications for using the EZ-Magna RIP Kit (Millipore) in accordance with the manufacturer’s instructions.

### Public expression profiles

Human pseudogene list is obtained from NCBI gene. Gene expression profiling datasets between HCC tumour tissues and normal tissues, including GSE84005, GSE76297, GSE6404, GSE54236, and GSE5975 were downloaded from GEO and TCGA. The expression data of HCC cancer cell lines was downloaded from CCLE.

### Dual luciferase reporter assay

A dual luciferase reporter assay was performed as previously described with slight modifications. Briefly, the treated HCC cells were lysed, and the activities of the firefly and Renilla luciferases were analysed using a dual luciferase assay kit (Promega, Madison, WI, USA) according to the manufacturer’s instructions. All reporter gene assays were performed in triplicate and were repeated twice. The results are expressed as the means ± SEM.

### Plasmids

Luciferase reporter gene constructs containing the *RACGAP1P* and *RACGAP1 3’ UTR* region were first amplified by PCR with specific primers and then, the PCR products were cloned into pGL3-basic (Promega) digested by XbaI(NEB) using ClonExpress II One Step Cloning Kit (C112-02, Vazyme Biotech).

Full length or 3’UTR of *RACGAP1P* were amplified by PCR from DNA of Hep3B cells and were cloned into lentivirus vector pLVX-puro according to standard protocols. *RACGAP1P* and *RACGAP1* 3’UTR was also subcloned into the pMS2 vector for RIP analysis.

### Immunoblotting

Immunoblotting was performed as described previously. The sources of the primary antibodies were as follows: anti-RACGAP1 (Proteintech, 1:1,000), anti-DICER1 (Proteintech, 1:1,000), and anti-β-actin (Proteintech, 1:1,000), p-ERK1/2 (CST, 1:1,000), total ERK (CST, 1:1,000), Active Rho Detection kit (#8820), anti-mouse and anti-rabbit peroxidase conjugated secondary antibodies were purchased from Cell Signalling Technology (Danvers, MA).

### Statistical analysis

The two-tailed Student’s *t*-test was used to compare two independent groups of data. One-way analysis of variance (ANOVA) was used to analyze the significance among multi-groups. Chi-squared tests were applied to analyze the relationship between *RACGAP1P* expression and clinicopathological status. Pearson correlation analysis, Long-rank test were performed as indicated. *P* value < 0.05 was considered statistically significant in all cases. Asterisk (*) means *p* < 0.05, asterisks (**) means *p* < 0.01, asterisks (***) means *p* < 0.001. All statistical analyses were performed using the GraphPad Prism Software 5.0 or SPSS 16.0.

## Results

### Pseudogene *RACGAP1P* is consistently activated in early recurrent HCC and serves as a predictor of dismal outcome

**Fig. 1:**
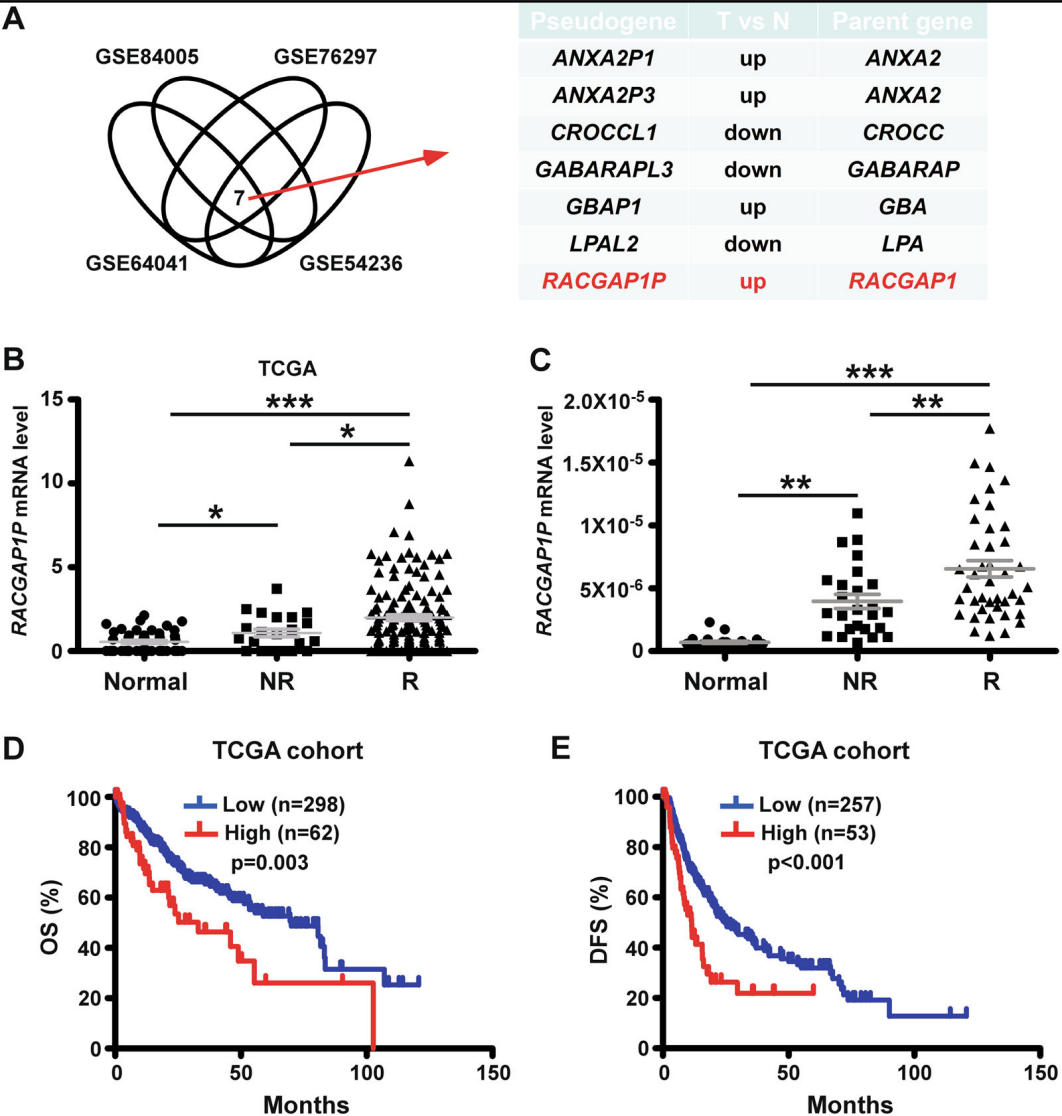
Pseudogene *RACGAP1P* is elevated in hepatocellular carcinoma (HCC) and is related to early HCC recurrence. **a** Venn diagram analysis of altered pseudogenes profiling in GSE84005, GSE76297, GSE6404, and GSE54236 datasets. **b** The *RACGAP1P* mRNA expression were analysed in normal liver tissues (*n* = 47), non-recurrent tumours (NR-HCC, *n* = 22) and early recurrent tumours (R-HCC, *n* = 141) from TCGA. The highest *RACGAP1P* mRNA expression were shown in human hepatocellular carcinoma early recurrent tissues. **c** The *RACGAP1P* mRNA expression were tested in human hepatocellular carcinoma tissues by QPCR, including normal liver tissues (*n* = 25), non-recurrent tumours (NR-HCC, *n* = 25), early recurrent tumours (R-HCC, *n* = 41). **d**, **e** Kaplan–Meier curve shows that the overall survival (OS) and disease-free survival (DFS) rate were significantly lower in the high *RACGAP1P* group in TCGA cohort. ***P < 0.001, **P < 0.01, *P < 0.05. B&C, the *p* values were calculated by one-way ANOVA. D&E, the *p* values were calculated by long-rank test

To evaluate the role of pseudogenes in HCC pathogenesis, we screened potential cancer-related pseudogenes, which were differently expressed between HCC samples and normal samples in 4 microarray datasets (GSE84005, GSE76297^17^, GSE64041^18^, GSE54236^19^) downloaded from Gene Expression Omnibus (GEO) and identified 7 candidates, of which 4 genes were upregulated and 3 genes were downregulated (Fig. 1a). We put our attention on *RACGAP1P* since its real gene, *RACGAP1*, was well-known to act as a oncogenic driver in HCC and importantly, played a key role in HCC early recurrence^6^. Similarly, assessment via data mining of RNA-seq data from The Cancer Genome Atlas (TCGA) database confirmed that *RACGAP1P* expression is significantly elevated in tumour tissues compared with non-tumour tissues. Of note, *RACGAP1P* expression was obviously upregulated in tumour from patients with early (<2 years) recurrent disease (HCC-R) compared with samples from patients with no obvious sign of recurrence within 4 years (HCC-NR) (Fig. 1b). The analogous pattern of *RACGAP1P* expression was further validated by QPCR (Fig. 1c). Moreover, in TCGA cohort, correlation analysis between *RACGAP1P* expression and clinicopathological characteristics shown that high level of *RACGAP1P* in primary tumours was significantly associated with the advanced T stage, clinical stage and abnormal AFP level (Table 1). Importantly, it was demonstrated that patients with high *RACGAP1P* expression had a significantly shorter overall (OS) and disease-free (DFS) duration compared with patients with low *RACGAP1P* expression (Fig. 1d, e). Taken together, these data sustain *RACGAP1P* as a novel prognostic factor in patients with HCC.

**Table 1:** Association of *RACGAP1P* expression with clinicopathological characteristics of HCC patients in the TCGA cohort

Characteristics	No. of patients	*RACGAP1P* expression		*p* value
		Low	High	
All patients	360	298	62	
Age				
Median	60			
Range	16–85			
≦60	173	138	35	
>60	187	160	27	0.146
Gender				
Male	244	205	39	
Female	116	93	23	0.367
T stage				
T1-T2	268	230	38	
T3-T4	91	67	24	0.008
N stage				
N0	247	204	43	
NX	112	93	19	0.918
M stage				
M0	264	214	50	
MX	96	84	12	0.152
Clinical stage				
I-II	252	217	35	
III-IV	87	64	23	0.007
Grade				
G1-G2	230	196	34	
G3-G4	130	102	28	0.103
AFP				
Normal	117	105	12	
Abnormal	152	123	29	0.046
Vascular invasion				
None	204	173	31	
Micro	89	78	11	
Macro	16	12	4	0.416
Cirrhosis				
No	131	114	17	
Yes	76	62	14	0.29
Inflammation extent				
No	116	102	14	
Yes	115	94	21	0.189
Virus(HBV or HCV)				
No	193	158	35	
Yes	149	124	25	0.744

### Pseudogene *RACGAP1P* modulates *RACGAP1* level in a ceRNA manner

**Fig. 2:**
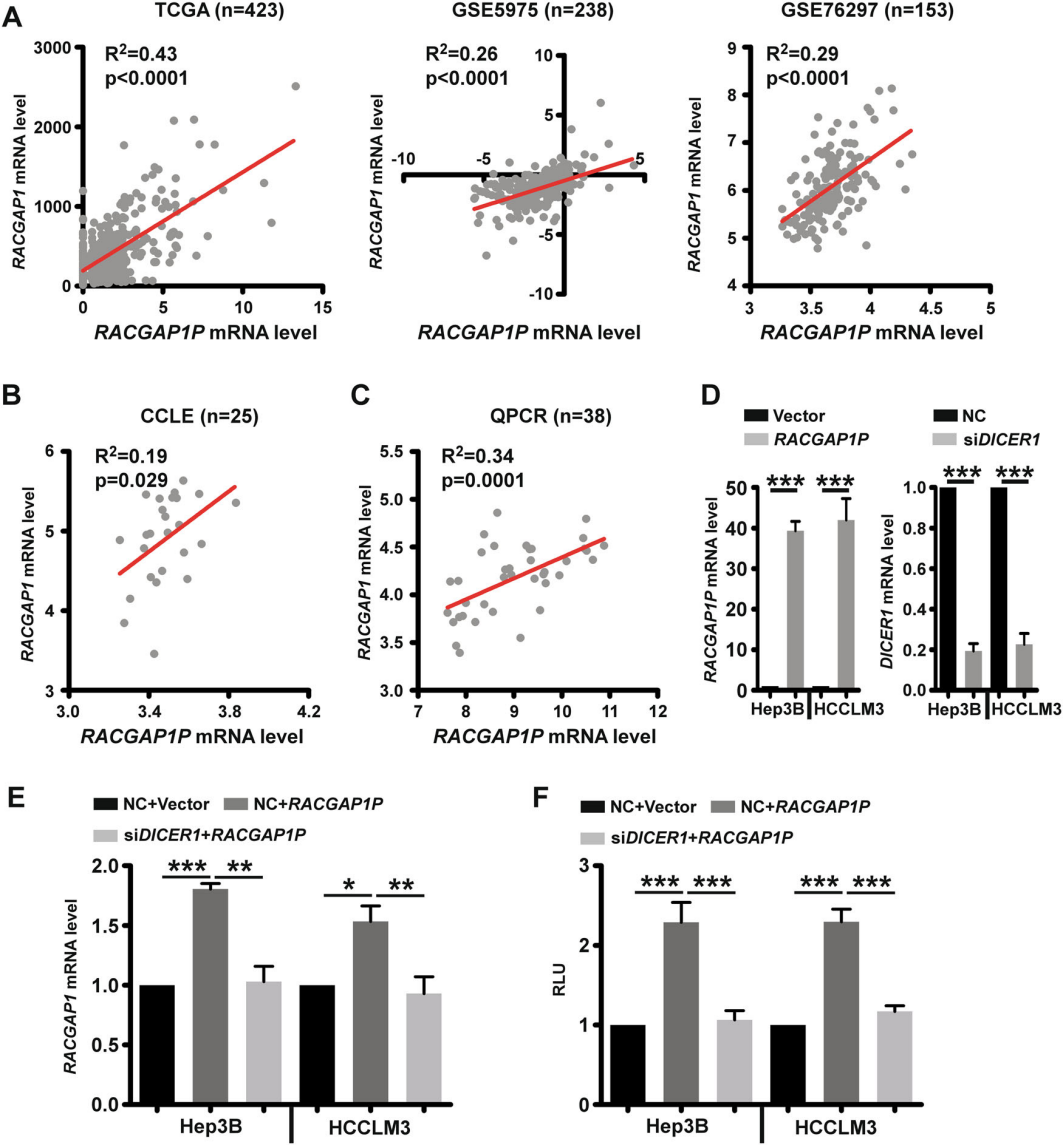
*RACGAP1P* is co-expressed with *RACGAP1* in HCC and regulates RACGAP1 expression dependent on mature miRNA. **a**, **b** Graphs showing a positive correlation between *RACGAP1P* and *RACGAP1* in HCC using public gene expression datasets, including TCGA (*n* = 423, *R*^2^ = 0.43, *p* < 0.0001), GSE5975 (*n* = 238, *R*^2^ = 26, *p* < 0.0001), GSE76279 (*n* = 153, *R*^2^ = 0.29, *p* < 0.0001) and CCLE (*n* = 25, *R*^2^ = 0.19, *p* = 0.029). A, HCC clinical specimens, B, HCC cell lines. **c** The co-expression between RACGAP1P and RACGAP1 mRNA level was measured in 38 primary HCC tissues via QPCR (*n* = 38, *R*^2^ = 0.34, *p* = 0.0001). **d** The efficiency of RACGAP1P overexpression and DICER1 knockdown in HCC cell lines were determined by QPCR. **e** In Hep3B and HCCLM3 cells, RACGAP1P upregulated RACGAP1 mRNA levels in a DICER1 dependent manner. **f** RACGAP1P increased RACGAP1 luciferase reporter activity in a DICER1 dependent manner. Error bars represent means ± SD. ***P < 0.001, **P < 0.01, *P < 0.05, the *p* values were calculated by the Student’s *t*-test

Recently, the competitive endogenous RNAs (ceRNA) hypothesis has been raised as a novel layer of gene expression modulation. The hypothesis proposed that RNA molecules share miRNA response elements (MREs) and sequester common microRNAs (miRNAs), thereby regulating expression between each other^20–22^. It is not difficult to speculate that pseudogenes may serve as ceRNAs for their parent genes, considering the high sequence similarity. To test the hypothesis that pseudogene *RACGAP1P* may regulate the expression of its parent gene as a ceRNA, we firstly assessed the correlation of *RACGAP1P* and *RACGAP1* via mRNA expression data derived from the TCGA, GEO (GSE5975, GSE76297) and Cancer Cell Line Encyclopaedia (CCLE) and found the co-expression patterns between *RACGAP1P* and *RACGAP1* in primary HCC tissues, as well as HCC cell lines (Fig. 2a, b). Then, we perform QPCR assay to validate the significantly positive correlation in HCC samples (*R*^2^ = 0.34, *P* = 0.0001; Fig. 2c). Next, to experimental examine whether *RACGAP1P* regulate the expression of *RACGAP1*, and whether the effect of the *RACGAP1P* on *RACGAP1* expression was in a miRNA-dependent manner, we suppressed *DICER1*, a ribonuclease critical for miRNA biogenesis, in Hep3B and HCCLM3 cells using a specific siRNA (Fig. 2d, Supplementary Fig. 1a) and found that ectopic expression of *RACGAP1P* in wild type cells elevated the mRNA level of *RACGAP1* but not in *DICER1*-KD cell lines (Fig. 2e). Similarly, *RACGAP1 3’UTR* luciferase reporter activity was significantly increased in *DICER1-*WT cells with *RACGAP1P* overexpression whereas these effects were abrogated in *DICER1*-KD cells (Fig. 2f). Thus, the *RACGAP1* pseudogene-induced effects on the *RACGAP1* level are 3’UTR and miRNA dependent in human HCC.

### Identification of the common miRNAs sharing between *RACGAP1P* and *RACGAP1*

**Fig. 3:**
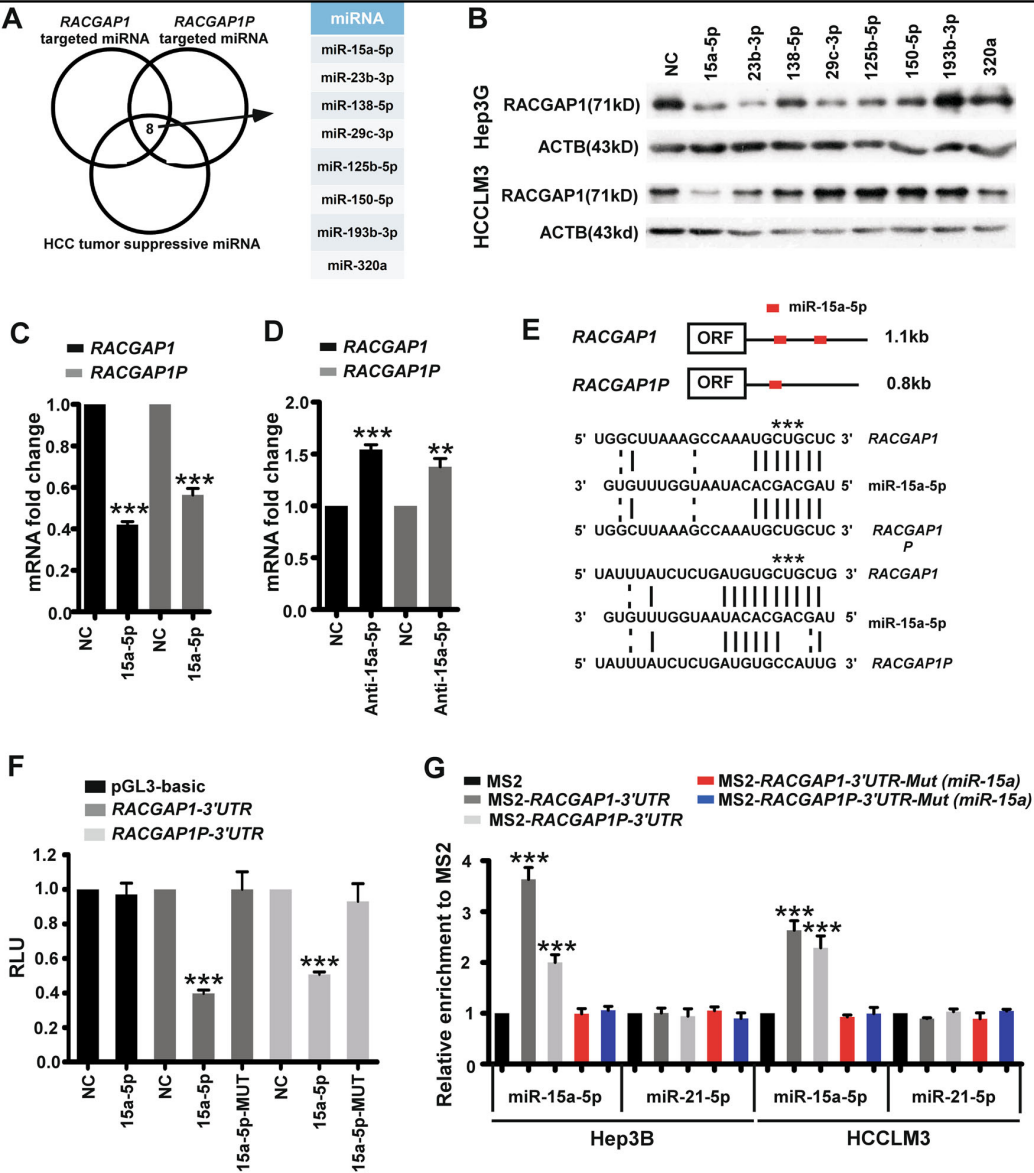
*miR-15-5p* targets both *RACGAP1P* and *RACGAP1*. **a** Eight candidate miRNAs were identified to be associated with *RACGAP1P* and *RACGAP1* in HCC from Venn diagram analysis of three datasets, including tumour suppressor miRNAs dataset in HCC, the miRNAs datasets predicted to bind *RACGAP1P* and *RACGAP1*. **b** The 8 candidate miRNAs differentially regulated the RACGAP1 protein expression in Hep3B and HCCLM3 cells shown by WB. **c** QPCR analysis of *RACGAP1P* and *RACGAP1* with *miR-15a-5p* mimics. **d** QPCR analysis of *RACGAP1P* and *RACGAP1* with *miR-15a-5p* antagomics. **e** *miR-15-5p* seed sequence is shown along with the binding sites in *RACGAP1P* and *RACGAP1* (shown by red box), and the mutated *miR-15-5p* binding sites (shown by star). **f** luciferase reporter assays verified the miR-15a-5p binding sites in 3’UTR of *RACGAP1P* and *RACGAP1*. **g** MS2-RIP strategy used to confirm endogenous *miR-15a-5p* binding to *RACGAP1P* and *RACGAP1*. Error bars represent means ± SD. ***P < 0.001, **P < 0.01, Student’s *t*-test

The finding that the pseudogene *RACGAP1* mediates its effect through 3’UTR and mature miRNAs suggests that it may function as a ceRNA. To further elucidate which of the miRNAs targeted both *RACGAP1P* and *RACGAP1* to mediate the observed crosstalk, we overlaid the miRNAs predicted to target *RACGAP1* and *RACGAP1P* in RegRNA2.0^23^ and previously reported HCC tumour suppressive miRNAs^24^ and finally obtained 8 candidate miRNAs including *miR-15a-5p*, *miR-23b-3p*, *miR-138-5p*, *miR-29c-3p*, *miR-125b-5p*, *miR-150-5p*, *miR-193b-3p*, *miR-320a* (Fig. 3a). Then, Hep3B and HCCLM3 cells were delivered with these 8 potential miRNA mimics, respectively, and the expression of RACGAP1 was detected by western blot analysis. As shown in Fig. 3b, only *miR-15a-5p* was validated to significantly repress protein abundance of RACGAP1 in both cell lines. Therefore, *miR-15a-5p* was selected for further analysis and QPCR results revealed that miR-15a-5p overexpression strongly inhibited the mRNA level of *RACGAP1* and *RACGAP1P* (Fig. 3c). Conversely, inhibition of *miR-15a-5p* using *miRNA* antagomirs could upregulated mRNA levels of both *RACGAP1* and *RACGAP1P* (Fig. 3d).

To further investigate whether *miR-15a-5p* could target both *RACGAP1* and *RACGAP1P*, we performed the prediction of *miR-15a-5p* MREs for *RACGAP1* and *RACGAP1P* transcripts and found two *miR-15a-5p* binding sites in *RACGAP1 3’UTR* and one in *RACGAP1P 3’UTR* (Fig. 3e). Then, we artificially introduced mutation of *miR-15a-5p* seed regions in luciferase reporters to disrupt the miRNA binding. The results of luciferase reporter assay demonstrated that overexpression of *miR-15a-5p* significantly decreased luciferase reporter activity of *RACGAP1P-3’UTR* and *RACGAP1-3’UTR* whereas the inhibition effect of *miR-15a-5p* was rescued on luciferase reporter with miR-15a-5p binding sites mutations (Fig. 3f). To validate the direct binding ability of the predicted miRNA response elements on these transcripts, we performed an MS2-RIP analysis using the 3’UTR regions of *RACGAP1P* and *RACGAP1* as baits in Hep3B and HCCLM3 cells. Notably, *miR-15a-5p* was highly associated with the 3’UTRs of *RACGAP1P* and *RACGAP1*, and *miR-21-5p* as a control miRNA with no predicted MREs on *RACGAP1P* and *RACGAP1* bound neither 3’UTR. However, the binding of *miR-15a-5p* on *RACGAP1P* and *RACGAP1* 3’UTR was negated in MREs mutation group (Fig. 3g). All together, these findings indicated that the ceRNA crosstalk between *RACGAP1P* and *RACGAP1* may be mediated at least through *miR-15a-5p* in part.

### *RACGAP1P* is an oncogenic driver in HCC dependent on miRNA

**Fig. 4:**
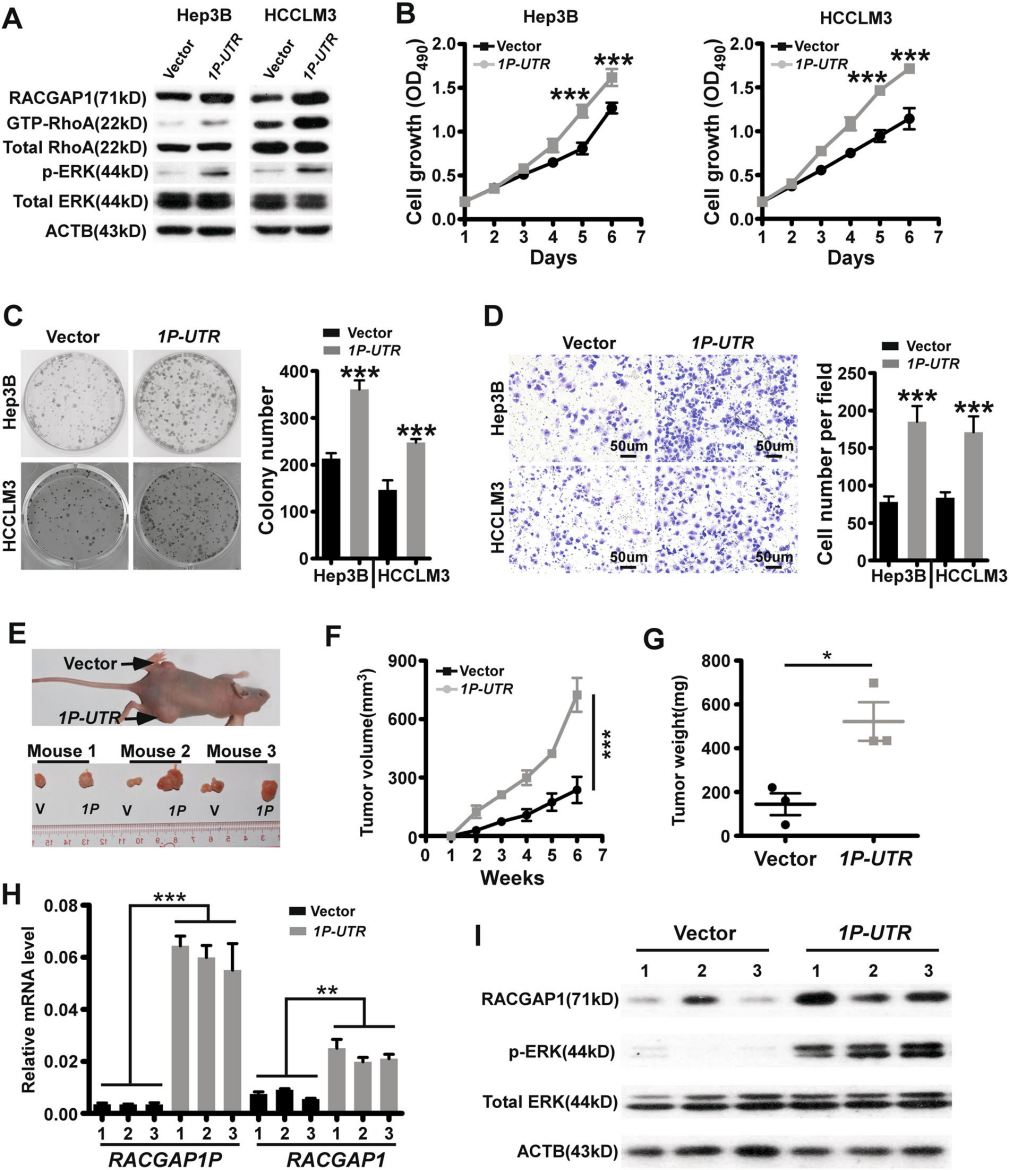
*RACGAP1P* regulates the growth and migration of HCC cells via RhoA/ERK signalling pathway. **a** RACGAP1 expression, RhoA and ERK activation were measured by WB after *RACGAP1P-3’UTR* overexpression in Hep3B and HCCLM3 cells. **b** MTT assays shows that *RACGAP1P-3’UTR* overexpression promoted HCC cells growth. **c** Colony formation assays shows that *RACGAP1P-3’UTR* overexpression enhanced the proliferation of HCC cancer cells. Left: Representative images of colony formation. Right: Statistical results of colony number. **d** Transwell assays indicates that *RACGAP1P-3’UTR* overexpression augmented the migration ability of HCC cells. Left: Representative images of migrated cells. Right: statistical results of migrated cells. **e**–**f** Subcutaneous tumour growth in mice inoculated with *RACGAP1P-3’UTR* overexpression Hep3B cells or or control cells. **e** Photos of xenograft tumours, (**f**) growth curve of tumours, and (**g**) statistical analysis of tumour weight growth over 6 weeks. **h** QPCR reslults show that higher mRNA level of *RACGAP1P* and *RACGAP1* in *RACGAP1P-3’UTR* overexpression xenograft tumours. **i** Western bloting indicates that the protein level of RACGAP1 and p-ERK were higher in *RACGAP1P-3’UTR* overexpression xenograft tumours. Error bars represent means ± SD. *P < 0.05, **P < 0.01, ***P < 0.001, the Student’s *t*-test

**Fig. 5:**
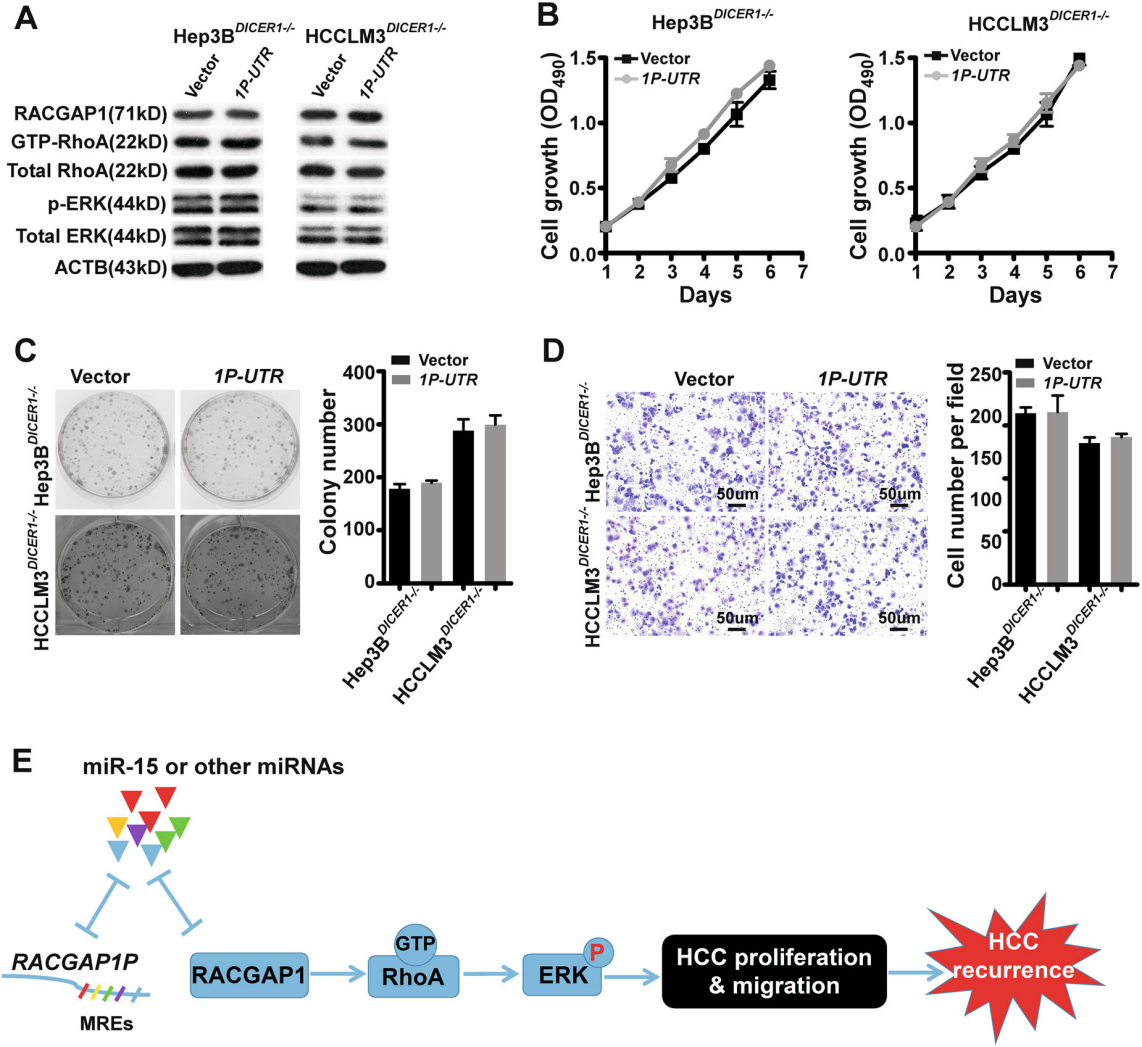
The oncogenctic activity of RACGAP1P depends on miRNA. **a** RACGAP1 expression, RhoA and ERK activation were detected by immunoblotting after *RACGAP1P-3’UTR* overexpression treatment in HCC cells with *DICER1* knockout. **b**–**d** The cell growth and migration was inhibited in *RACGAP1P* overexpression cells with *DICER1* depletion. **b** MTT assays. **c** Colony formation assays. **d** Transwell assays. **e** Diagram depicting the underlying mechanism of *RACGAP1P* contributing to HCC recurrence. *RACGAP1P* activates RhoA/ERK signalling axis by modulating *RACGAP1P/miR-15a/RACGAP1* ceRNA network

Considering increased RACGAP1 expression drives the tumourigenesis and progression of numerous types of cancer^25–28^, especially HCC, we determined whether aberrant control of RACGAP1 by *RACGAP1P* overexpression enhanced oncogenic transformation of HCC cells. Indeed, Stably exogenous expression of *RACGAP1P* 3’UTR in Hep3B and HCCLM3 resulted in a elevation of RACGAP1 protein, GTP-binding activity of RhoA, and ERK phosphorylation, indicating the activation of the RhoA/ERK pathway (Fig. 4a). These molecular observations were accompanied by growth promotion and a significant increase in the number of colonies, as well as high migration ability (Fig. 4b–d). Furthermore, to determine whether *RACGAP1P* affects tumour growth in vivo, we utilised a subcutaneous xenograft mouse model which were inoculated with Hep3B cells stably transfected with control vector or *RACGAP1P 3’UTR*. All of the mice developed tumours at the injection sites. However, the average size and weight of tumours generated by *RACGAP1P 3’UTR*-expressing cells were significantly larger than those generated from control cells (Fig. 4e–g). QPCR analysis confirmed that the average expression of *RACGAP1P* and *RACGAP1* was much higher in *RACGAP1P 3’UTR over*expressing tumour tissues compared with control tumours (Fig. 4h). Finally, immunoblotting showed that expression of RACGAP1 and phosphorylated ERK was higher in *RACGAP1P 3’UTR*-expressing tumours than control tumours (Fig. 4i). Importantly, the cancer cell proliferation and migration promotion effects were abrogated in *DICER1*-knock out HCC cells (Fig. 5a–d, Supplementary Fig. 1b), supporting the notion that the 3’UTR of *RACGAP1P* requires mature miRNAs for its function towards *RACGAP1*. Taken together, our results demonstrate that *RACGAP1P*, operating as a ceRNA, activates RACGAP1/RhoA/ERK pathway to favour malignant phenotypes of HCC (Fig. 5e).

### *RACGAP1P* crosstalks with *RACGAP1* and associates with poor prognosis in various human cancers

**Fig. 6:**
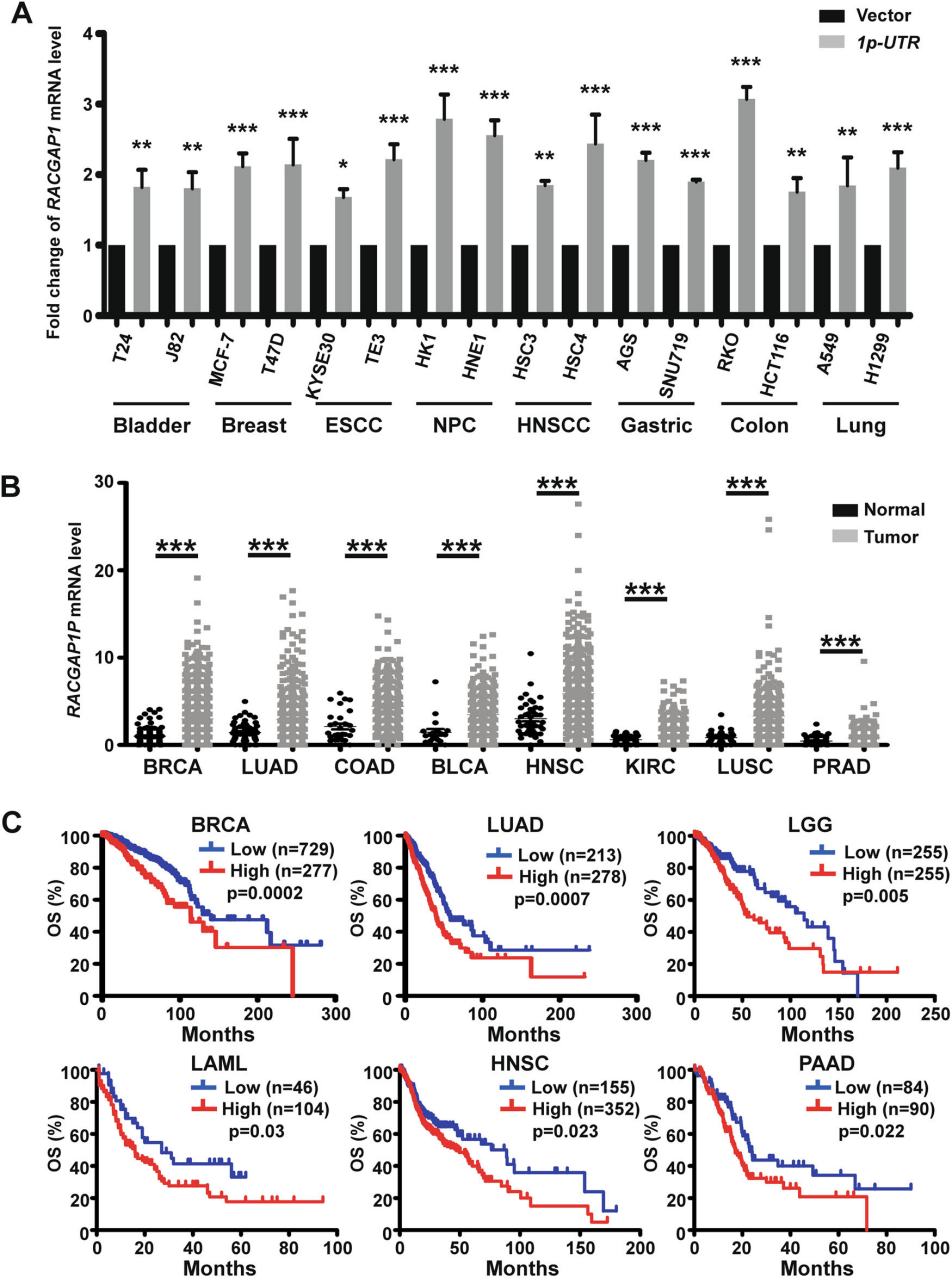
*RACGAP1P* is elevated in multiple human cancers and correlates with poor outcome. **a** QPCR analysis indicates that overexpression of *RACGAP1P 3’UTR* induced the expression of RACGAP1 in various human cancer cell lines. **b** The mRNA Level of *RACGAP1P* is significantly upregulated in 8 types of human cancer. **c** High expression of *RACGAP1P* was related to worse overall survival (OS) in 6 types of cancer. BLCA bladder urothelial carcinoma, BRCA breast invasive carcinoma, CESC cervical squamous cell carcinoma and endocervical adenocarcinoma, COAD colon adenocarcinoma, HNSC head and neck squamous cell carcinoma, KIRC kidney renal clear cell carcinoma, LUAD lung adenocarcinoma, LUSC lung squamous cell carcinoma, PAAD pancreatic adenocarcinoma, PRAD prostate adenocarcinoma, LGG low grade glima, AML acute myeloid leukaemia. Error bars represent means ± SD. *P < 0.05, **P < 0.01, ***P < 0.001, the *p* values were calculated by Student’s *t*-test or long-rank test

To figure out whether the ceRNA regulation between *RACGAP1P* and *RACGAP1* is HCC specific or common in pan-cancers, the analysis of data from TCGA was performed and found the highly positive correlation between *RACGAP1P* and *RACGAP1* in various types of cancer (Supplementary Fig. 2). In addition, exogenous overexpression of *RACGAP1P 3’UTR* in different human cancer cell lines could induce *RACGAP1* expression showed by QPCR results (Fig. 6a). Moreover, the mRNA level of *RACGAP1P* was significantly upregulated in a great many human cancers compared with the normal tissues (Fig. 6b). Further, a high level of *RACGAP1P* indicated the shorter overall survival in pan-cancers, such as BRCA, LUAD, LGG, LAML, HNSC, PAAD (Fig. 6c). Thus, high *RACGAP1P* level in human cancers significantly correlated with poor outcome.

## Discussion

Early recurrence is the major obstacle in achieving long-term survival outcomes for the treatment of HCC via operative treatment. Our knowledge of the underlying cellular and molecular pathways driving HCC early relapse is rather finite. Previous studies have addressed the clinical implication of gene expression profiling in predicting the early recurrence of human HCC^29^. However, there is pretty limited information about ncRNA, especially pseudogenes involved in HCC early relapse. In the present study, we identified a novel liver cancer early recurrence associated lncRNA *RACGAP1P*, which is significantly upregulated in liver cancer tissues and mainly expressed much higher in recurrent HCC. Higher expression of *RACGAP1P* was correlated with late T stage, advanced clinical stage and abnormal AFP level. Moreover, increased *RACGAP1P* expression was associated with shorter OS and PFS of HCC patients. In vitro and in vivo assays demonstrated that ectogenic expression of *RACGAP1P* promoted cell proliferation and migration. These findings indicate that *RACGAP1P* has an oncogenic role in liver tumourigenesis and could be considered as a potential prognostic indicator for HCC.

RACGAP1, a member of GTPase activation protein family^30^, is the first gene reported to work as an independent informative biomarker for HCC early recurrent, as well as its silencing mainly affected genes in an interactome clinically relevant to early relapse. Given the significant effects of RACGAP1 on cancer recurrence, the regulatory network related to RACGAP1 urgently needed to be explored. Genetic or epigenetic alterations may directly lead to the aberrant expression of cancer-related genes. Notably, data analysing of the TCGA data found that the *RACGAP1* locus undergoes copy number gains in 13.2% liver cancers and the methylation level in the promoter region is lower in cancer tissues than normal tissues and negatively associated with *RACGAP1* mRNA level. However, the copy number and the methylation level were no obvious difference between recurrent and non-recurrent HCC (Supplementary Fig. 3). Therefore, the regulatory mechanisms of increasing expression of RACGAP1 in early recurrent HCC still remained to be elucidated. Herein, it was interesting to observe that the expression of pseudogene *RACGAP1P* significantly correlated with its cognate gene *RACGAP1* expression and overexpression of *RACGAP1P* could increase the expression of *RACGAP1* dependent on miRNA. Moreover, *RACGAP1P* is consistently upregulated in early recurrent HCC samples. All above suggest *RACGAP1P* may involve in the elevation of *RACGAP1* during HCC early recurrence.

In recent years, ceRNA hypothesis provides a new crosstalk dimension between RNA moleculars that serve as miRNA sponges to regulate the level of the genes which harbour the common miRNA binding sites. Given the high sequence similarity between pseudogenes and their parent genes, pseudogenes are “perfect decoys” for their ancestral genes, critically impacting their parent protein-coding genes via the ceRNA network^31,32^. Based on the central of ceRNA hypothesis that miRNAs play as communication language, we identified *miR-15a-5p*, which is a well-known tumour suppressor in the pathogenesis of HCC, could target both *RACGAP1P* and *RACGAP1*. And the direct interaction between *miR-15a-5p* and these paired genes was confirmed by dual luciferase reporter assay and MS2-RIP assay, indicating that *RACGAP1P* regulates *RACGAP1* expression at least partially through competitive binding with *miR-15a-5p* as a ceRNA. Similarly, the studies by the groups of Shen et al., Sharp et al. and Stoffel et al. focused on ceRNA regulation that is mediated by a single miRNA. Importantly, ceRNA pairs in general, and gene/pseudogene pairs in particular, share numerous miRNAs and it is reported that ceRNA crosstalk is enhanced when it is mediated by more miRNAs^33,34^. Therefore, more shared miRNAs that being present at crosstalk-favoring levels needed to be identified.

Pseudogenes could exert critical functions in biological processes and their dyregulation may conduce to the occurrence and progression of diseases^12,35^. Herein, our study establishes the *RACGAP1P* as a potent proto-oncogene that can activate RACGAP1/Rho/ERK axis to elicit malignant phenotypes, such as cell growth and migration. Mechanistically, *RACGAP1P* works as a ceRNA to compete with *miR-15a-5p* for binding to the 3’UTR region to relieve the inhibitory effects of *miR-15a-5*p on RACGAP1 expression, whereas MRE mutations impair the regulation of RACGAP1 by *RCGAP1P*. These findings provide strong evidence in support of a key role for *RACGAP1P* in the tumourigenesis of liver cancer. It is possible that *RACGAP1P* elicits its effects through more than one mechanism or pathway, so whether the oncogenic activity of *RACGAP1P* also requires additional ceRNA targets or non-ceRNA-related mechanisms will be the focus of future studies.

## Supplementary information

Supplemental information

## References

[1] Ulahannan SV (2014). Earlier presentation and application of curative treatments in hepatocellular carcinoma. Hepatology.

[2] Li W (2013). Accumulation of the mutations in basal core promoter of hepatitis B virus subgenotype C1 increase the risk of hepatocellular carcinoma in Southern China. Int. J Clin. Exp. Pathol..

[3] Hoshida Y (2009). Risk of recurrence in hepatitis B-related hepatocellular carcinoma: impact of viral load in late recurrence. J. Hepatol..

[4] El-Serag HB (2011). Hepatocellular carcinoma. N. Engl. J. Med..

[5] Feng GS (2012). Conflicting roles of molecules in hepatocarcinogenesis: paradigm or paradox. Cancer Cell.

[6] Wang SM, Ooi LL, Hui KM (2011). Upregulation of Rac GTPase-activating protein 1 is significantly associated with the early recurrence of human hepatocellular carcinoma. Clin. Cancer Res..

[7] Chen J (2015). ECT2 regulates the Rho/ERK signalling axis to promote early recurrence in human hepatocellular carcinoma. J. Hepatol..

[8] Chen J (2016). The microtubule-associated protein PRC1 promotes early recurrence of hepatocellular carcinoma in association with the Wnt/beta-catenin signalling pathway. Gut.

[9] Balakirev ES, Ayala FJ (2003). Pseudogenes: are they “junk” or functional DNA?. Ann. Rev. Genet..

[10] Li W, Yang W, Wang XJ (2013). Pseudogenes: pseudo or real functional elements?. J. Genet. Genomics.

[11] Han L (2014). The Pan-Cancer analysis of pseudogene expression reveals biologically and clinically relevant tumour subtypes. Nat. Commun..

[12] Cooke SL (2014). Processed pseudogenes acquired somatically during cancer development. Nat. Commun..

[13] Wang L (2013). Pseudogene OCT4-pg4 functions as a natural micro RNA sponge to regulate OCT4 expression by competing for miR-145 in hepatocellular carcinoma. Carcinogenesis.

[14] Peng H (2015). Pseudogene INTS6P1 regulates its cognate gene INTS6 through competitive binding of miR-17-5p in hepatocellular carcinoma. Oncotarget.

[15] Kong Y (2017). Pseudogene PDIA3P1 promotes cell proliferation, migration and invasion, and suppresses apoptosis in hepatocellular carcinoma by regulating the p53 pathway. Cancer Lett..

[16] Gong C, Maquat L (2011). E. lncRNAs transactivate STAU1-mediated mRNA decay by duplexing with 3’ UTRs via Alu elements. Nature.

[17] Chaisaingmongkol J (2017). Common molecular subtypes among asian hepatocellular carcinoma and cholangiocarcinoma. Cancer Cell.

[18] Makowska Z (2016). Gene expression analysis of biopsy samples reveals critical limitations of transcriptome-based molecular classifications of hepatocellular carcinoma. J. Pathol. Clin. Res..

[19] Villa E (2016). Neoangiogenesis-related genes are hallmarks of fast-growing hepatocellular carcinomas and worst survival. Results from a prospective study. Gut.

[20] Salmena L, Poliseno L, Tay Y, Kats L, Pandolfi PP (2011). A ceRNA hypothesis: the Rosetta Stone of a hidden RNA language?. Cell.

[21] Karreth FA, Pandolfi P (2013). P. ceRNA cross-talk in cancer: when ce-bling rivalries go awry. Cancer Discov..

[22] Tay Y, Rinn J, Pandolfi PP (2014). The multilayered complexity of ceRNA crosstalk and competition. Nature.

[23] Chang TH (2013). An enhanced computational platform for investigating the roles of regulatory RNA and for identifying functional RNA motifs. BMC Bioinformatics.

[24] Wang D, Gu J, Wang T, Ding Z (2014). OncomiRDB: a database for the experimentally verified oncogenic and tumour-suppressive microRNAs. Bioinformatics.

[25] Mi S (2016). RNA-seq identification of RACGAP1 as a metastatic driver in uterine carcinosarcoma. Clin. Cancer Res..

[26] Imaoka H (2015). RacGAP1 expression, increasing tumour malignant potential, as a predictive biomarker for lymph node metastasis and poor prognosis in colorectal cancer. Carcinogenesis.

[27] Saigusa S (2015). Clinical significance of RacGAP1 expression at the invasive front of gastric cancer. Gastric Cancer.

[28] Ke HL (2013). Expression of RACGAP1 in high grade meningiomas: a potential role in cancer progression. J. Neuro-oncol..

[29] Wang SM, Ooi LL, Hui KM (2007). Identification and validation of a novel gene signature associated with the recurrence of human hepatocellular carcinoma. Clin. Cancer Res..

[30] Toure A (1998). MgcRacGAP, a new human GTPase-activating protein for Rac and Cdc42 similar to Drosophila rotundRacGAP gene product, is expressed in male germ cells. J. Biol. Chem..

[31] Poliseno L (2010). A coding-independent function of gene and pseudogene mRNAs regulates tumour biology. Nature.

[32] Karreth FA (2015). The BRAF pseudogene functions as a competitive endogenous RNA and induces lymphoma in vivo. Cell.

[33] Bosson AD, Zamudio JR, Sharp PA (2014). Endogenous miRNA and target concentrations determine susceptibility to potential ceRNA competition. Mol. Cell.

[34] Denzler R, Agarwal V, Stefano J, Bartel DP, Stoffel M (2014). Assessing the ceRNA hypothesis with quantitative measurements of miRNA and target abundance. Mol. Cell.

[35] Pink RC, Carter DR (2013). Pseudogenes as regulators of biological function. Essays Biochem..

