# Supplementary material for: Pseudogene RACGAP1P activates RACGAP1/Rho/ERK signalling axis as a competing endogenous RNA to promote hepatocellular carcinoma early recurrence
Source: Cell Death Dis. 2019 Jun 3;10(6):426. doi: 10.1038/s41419-019-1666-2 (PMC6546712; doi:10.1038/s41419-019-1666-2)
Supplement: Supplementary file 1 — Supplemental information [file 41419_2019_1666_MOESM1_ESM.doc]

**Supplemental information**

**
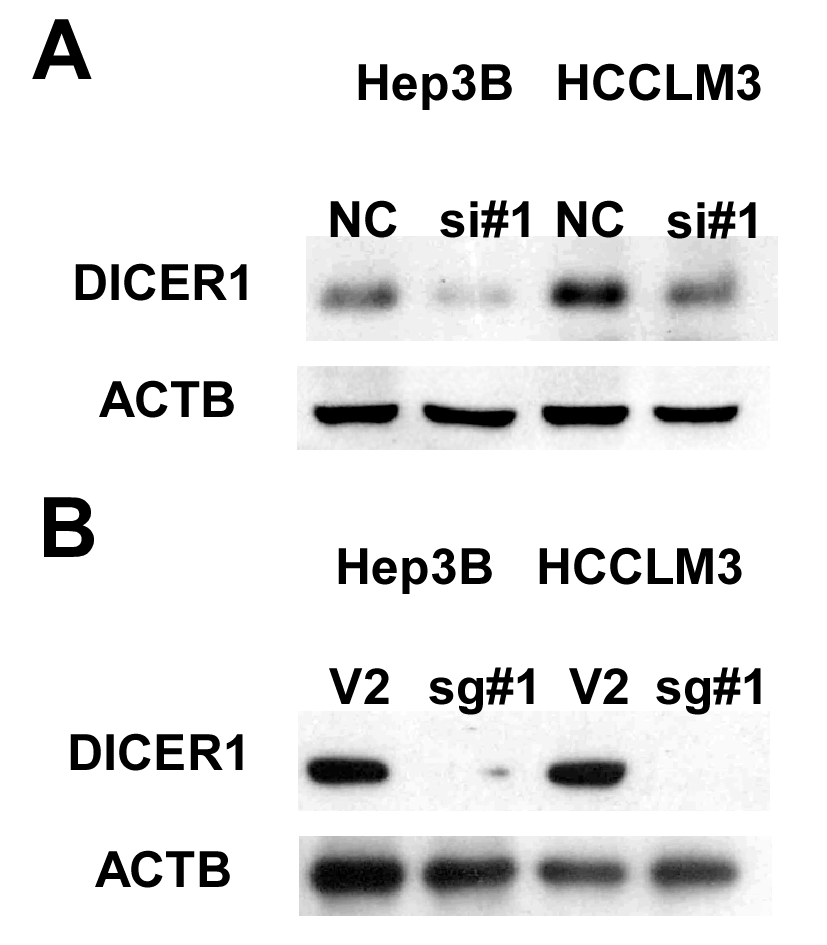
**

**Supplementary Figure 1 Western blotting confirms the efficiency of siRNA and sgRNA**


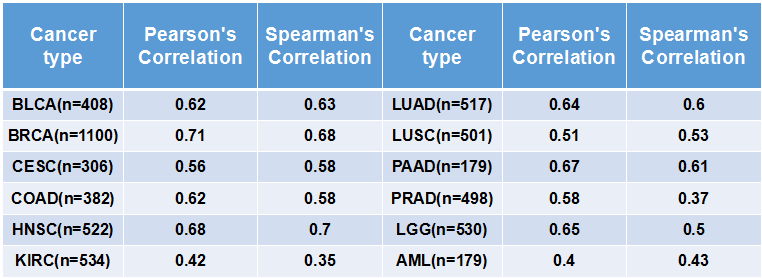


**Supplementary Figure 2 The correlation between *RACGAP1P*** **and *RACGAP1***

The table shows the positive correlation between *RACGAP1P* and *RACGAP1* mRNA level in 12 types of human cancer.

**
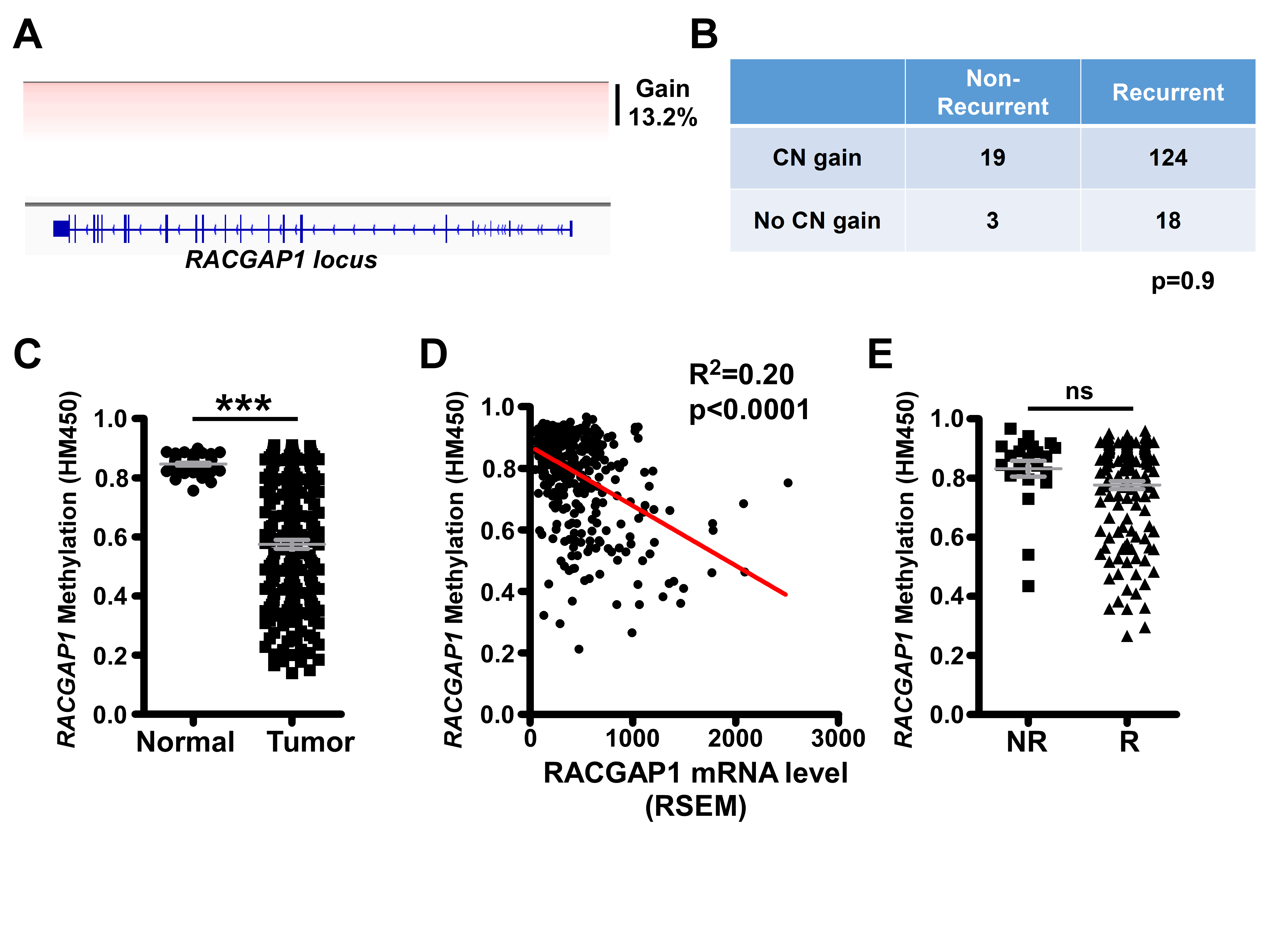
**

**Supplementary Figure 3 Copy number and the methylation level may not be [primarily](../../../../C:/Users/Administrator/AppData/Local/Youdao/Dict/Application/8.0.1.0/resultui/html/index.html#/javascript:;) responsible for the [aberrant](../../../../C:/Users/Administrator/AppData/Local/Youdao/Dict/Application/8.0.1.0/resultui/html/index.html#/javascript:;) expression of RACGAP1 in early recurrent HCC.**

1. The *RACGAP1* locus undergoes copy number gains in 13.2% liver cancers.
2. The copy number has no obvious difference between recurrent and non-recurrent HCC.
3. The methylation level in the *RACGAP1* promoter region is lower in cancer tissures than normal tissues.
4. The methylation level of *RACGAP1* was negatively associated with *RACGAP1* mRNA level.
5. The methylation level of *RACGAP1* has no obvious difference between recurrent and non-recurrent HCC.

Ns, nonsignificance, ***P < 0.001, the p values were calculated by the two-tailed Student’s t-test or [Chi-square](../../../../C:/Users/Administrator/AppData/Local/Youdao/Dict/Application/8.0.1.0/resultui/html/index.html#/javascript:;) [test](../../../../C:/Users/Administrator/AppData/Local/Youdao/Dict/Application/8.0.1.0/resultui/html/index.html#/javascript:;).
